# Lightening the Load: Generative AI to Mitigate the Burden of the New Era of Obesity Medical Therapy

**DOI:** 10.2196/58680

**Published:** 2024-11-14

**Authors:** Elizabeth R Stevens, Arielle Elmaleh-Sachs, Holly Lofton, Devin M Mann

**Affiliations:** 1 Department of Population Health, New York University Grossman School of Medicine, 227 e 30th st., New York, NY, 10016, United States, 1 646-501-2558; 2 Department of Medicine, New York University Grossman School of Medicine, New York, NY, United States; 3 Family Health Centers, New York University Langone Health, Brooklyn, NY, United States; 4 Department of Surgery, New York University Grossman School of Medicine, New York, NY, United States; ^5^MCIT Department of Health Informatics, New York University Langone Health, New York, NY, United States

**Keywords:** obesity, artificial intelligence, AI, clinical management, GLP-1, glucagon-like peptide 1, medical therapy, antiobesity, diabetes, medication, agonists, glucose-dependent insulinotropic polypeptide, treatment, clinician, health care delivery system, incretin mimetic

## Abstract

Highly effective antiobesity and diabetes medications such as glucagon-like peptide 1 (GLP-1) agonists and glucose-dependent insulinotropic polypeptide/GLP-1 (dual) receptor agonists (RAs) have ushered in a new era of treatment of these highly prevalent, morbid conditions that have increased across the globe. However, the rapidly escalating use of GLP-1/dual RA medications is poised to overwhelm an already overburdened health care provider workforce and health care delivery system, stifling its potentially dramatic benefits. Relying on existing systems and resources to address the oncoming rise in GLP-1/dual RA use will be insufficient. Generative artificial intelligence (GenAI) has the potential to offset the clinical and administrative demands associated with the management of patients on these medication types. Early adoption of GenAI to facilitate the management of these GLP-1/dual RAs has the potential to improve health outcomes while decreasing its concomitant workload. Research and development efforts are urgently needed to develop GenAI obesity medication management tools, as well as to ensure their accessibility and use by encouraging their integration into health care delivery systems.

## Introduction

Highly effective antiobesity and diabetes medications such as glucagon-like peptide 1 (GLP-1) agonists have ushered in a new era of treatment of these highly prevalent, morbid conditions that have increased across the globe over the past few decades. It is estimated that by 2030 nearly 30 million people in the United States will be on GLP-1 or glucose-dependent insulinotropic polypeptide/GLP-1 (dual) receptor agonists (RAs; henceforth referred to as GLP-1/dual RA) medications. Currently, their use is throttled by limited availability and insurance coverage challenges. As these issues resolve, their widespread use will trigger an even larger bottleneck—the substantial clinical management burden driven by the frequent communication, titration, and administrative interactions required to successfully manage obesity and related conditions using these important new medications. Indeed, health care providers (HCPs) and their practices have already begun to experience the strain of managing the high demand for weight loss medications. Relying on existing systems and resources to address the oncoming rise in GLP-1/dual RA use will be insufficient. Generative artificial intelligence (GenAI) has the potential to offset the clinical and administrative demands associated with the management of patients on these medication types. Research and development efforts are urgently needed to develop GenAI GLP-1/dual RA medication management tools, as well as to ensure their accessibility and use by encouraging their integration into health care delivery systems.

## The High Burden of Obesity Medications

When an HCP chooses to prescribe a GLP-1/dual RA to their patient, they are embarking on a months-long journey of clinical or administrative burden greater than most common chronic disease medications. A clinical team will be tasked with regularly balancing weight loss goals, hemoglobin A_1c_ targets, and side effects; continuously evaluating whether to continue to titrate up (or down) the medication until a maintenance dose is achieved. Moreover, HCPs will likely be faced with navigating insurance preauthorizations and fielding patient calls and messages about side effects, while searching for alternative pharmacies or bridging medications to address medication shortages.

On a small scale, this may be manageable, but as the number of patients on GLP-1/dual RAs expands to accommodate the 42% of Americans with obesity [1], it is unsustainable. With clinical practices already overburdened by administrative workload and HCPs at high risk for burnout [2], it is unreasonable to assume that the additional labor demands to manage patients on GLP-1/dual RAs could be handled by the existing workforce or that a health care system could feasibly hire enough additional personnel to meet this demand. To address the potential wave of future patients on GLP-1/dual RAs, tools are needed to reduce communication and administrative burden, allowing HCPs to focus on more complex patient care.

## GenAI Role in Medication Management

GenAI may represent an opportunity to automate many of the low-complexity, high-burden GLP-1/dual RA management tasks. As compared to previous iterations of artificial intelligence (AI), the technical functionality of GenAI allows for the creation of content, addressing numerous aspects of care management tasks that were previously impossible or overly burdensome to automate. Specifically, through the use of recurrent neural networks [3], generative adversarial networks [4], and large language models [5] with natural language processing [6] capabilities, GenAI has an inherent flexibility to combine heterogeneous sources of data to generate summaries, perform calculations, and create original content, including the production of potentially impactful metrics to improve clinical decision-making [7-10]. Furthermore, advancements in natural language understanding research have enabled the design of AI-driven chatbots—conversational agents that mimic human interaction through written, oral, and visual forms of communication with a user [11,12]. AI chatbots can learn from previous interactions, offering a more personalized, engaging, and on-demand user experience to support health behaviors [13,14]. The addition of GenAI functionality to AI-driven chatbots further improves the chatbot’s ability to respond dynamically. In these ways, the capabilities of GenAI extend its potential functionality well beyond a single algorithm for medication titration.

Indeed, while still an emerging technology, GenAI has shown itself to be a potentially effective tool for patient medication and care management in the areas of diabetes insulin management, hypertension, and weight management. In the form of chatbots, AI has demonstrated its use to facilitate the collection of patient data, reduce HCP message burden [14], and deliver health coaching for adults with overweight and obesity [12], producing similar results to those expected from in-person lifestyle interventions [15]. AI chatbots have also demonstrated the potential integration of wearable device data and messaging platforms for the creation of personalized intervention messaging [16]. GenAI-generated responses to patient questions have even been shown to be perceived as higher quality, more empathetic, and have greater clinical decision support accuracy than physician responses [17,18]. GenAI is also powering new “ambient clinical documentation” tools that effectively transform patient-clinician conversations into medical documentation [19].

Through the synthesis of patient data, clinical guidelines, and information databases, GenAI can provide effective and accurate clinical decision support and patient intervention, and even pharmacist-validated medication management [20]. For example, a voice-based conversational GenAI application effectively provided an autonomous real-time remote patient intervention for basal insulin management among patients with type 2 diabetes by incorporating HCP-selected titration algorithms and emergency protocols (parameters) for hypoglycemia and hyperglycemia based on daily patient reports of insulin dose and blood sugar value. This intervention led to significantly improved insulin management as compared to standard care [21]. Similarly, GenAI has revealed promise as a potential solution to the high burden incurred in remote patient monitoring for hypertension. By creating a GenAI-powered messaging platform for patient interactions, and integrating GenAI-created smart summaries into the electronic health record (EHR), these tools assisted in the management of the large volume of incoming data and have the potential to enhance both patient and HCP-facing tasks associated with digital health care for hypertension management [22]. These examples highlight how GenAI tools may be capable of supporting more efficient GLP-1/dual RA dose titration and increasing patient engagement without significantly increasing HCP workload.

**Figure 1.**
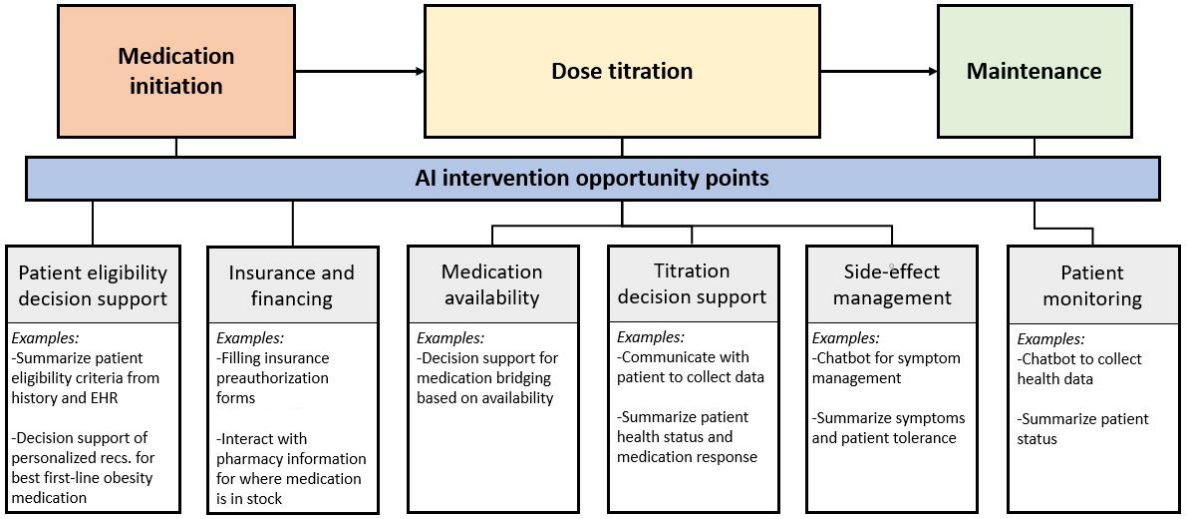
GLP-1/dual RA medication management workflow and example opportunities for GenAI intervention. AI: artificial intelligence; EHR: electronic health record; GenAI: generative artificial intelligence; GLP-1: glucagon-like peptide 1; RA: receptor agonist.

Similar to these example cases, the management of GLP-1/dual RA medications requires patient engagement, as well as the collation of information from patients themselves, medical records, and clinical guidelines. Furthermore, GLP-1/dual RA management can be subjective with nuance in the interpretation of patient symptom tolerability and thus requires greater use of clinical judgment as opposed to hard and fast rules or cutoffs. GenAI has the potential to address multiple aspects of GLP-1/dual RA management, including streamlining patient-HCP communication, giving HCPs recommendations on optimal dose titration, and providing prescribing guidance based on nonclinical factors such as insurance coverage and medication availability (Figure 1). Through its inherent flexibility to incorporate multiple data sources, GenAI can interpret patient natural language responses regarding side effects and weight-loss goals, as well as incorporate information living in the EHR and other databases such as patient characteristics including weight changes, medical history, current medication dose, blood sugar levels, and insurance status [7]. This enables GenAI to provide a broad range of GLP-1/dual RA management services from personalized guidance for patients on the management of side effects to clinical advisement to optimize dose titrations.

## Health Care System Integration of GenAI Interventions

Scaling the effective management of GLP-1/dual RAs, however, cannot be achieved through stand-alone development of GenAI tools, bots, and algorithms—they must be deeply integrated into the health care delivery system. This requires careful EHR and clinical workflow integration; a “last-mile” problem that most health care startups and innovators avoid until the end of their product development journey. While this may be consistent with their business plan, it has repeatedly led to low penetration of these potentially valuable digital tools. GenAI-assisted GLP-1/dual RA management will need early and deep clinical and EHR integration to disrupt this pattern.

To achieve clinical integration, however, there will be several privacy, cost, implementation, and ethical challenges that must be considered [23]. First, as with other forms of digitization of health care, the use of AI may introduce additional data privacy concerns regarding data storage, sharing, and use in model training [24]. Consequently, accommodations will need to be made to house and maintain any patient data, and the AI models being used, on internal firewall-protected servers, as opposed to externally hosted AI platforms [17].

The cost of integrating GenAI into clinical practice is also not insubstantial. In addition to costs associated with setting up and maintaining additional secure servers to house data, there are costs associated with each GenAI interaction. Depending on the task demanded, a sequence of several back-end prompts is likely required to achieve the desired outcome, with each prompt costing a multitude of “tokens” (ie, the basic units of text or code GenAI uses to process and generate language) and the use of each token coming at a monetary cost [25]. Moreover, each use of GenAI comes with an additional inference cost due to energy consumption, which can overtake the energy costs of training a GenAI model with high volumes of use [26].

To promote the successful implementation of GenAI products into clinical practice, usability, workflow integration, and user trust must be considered. Although presumptions have been made that the user-friendly, adaptable, and rapidly iterative aspects of GenAI will improve efficiency, productivity, and quality in ways not achieved with previous technologies [27], the deployment of GenAI interventions must be cognizant of clinical workflows, current technology integrations, and be designed with the user needs in mind [28]. Furthermore, the use of AI technologies in clinical care is not universally trusted by HCPs and patients [29,30], suggesting that substantial training and trust-building efforts will be required to improve acceptability and gain universal adoption.

Furthermore, there remain numerous ethical concerns associated with relying on GenAI in clinical care, including the potential exacerbation of disparities in health equity. Some ethical issues are associated with the technological aspects of GenAI functionality including the potential impact of algorithmic and language bias built into the training data used to create GenAI models, and how the reliability of models, including their potential for AI hallucinations, may impact clinical safety [31,32]. To address these types of concerns, health care institutions are likely to need a governance committee to oversee GenAI implementation, detail policies around data protection and data management practices, and thoroughly test GenAI models prior to allowing them for clinical use [33].

Digital health equity is another ethical consideration that will need to be addressed early and often in the development and deployment of GenAI-enabled clinical care, such as GLP-1/dual RA management tools. Due to structural inequities of access to insurance coverage, digital tools, and digital literacy, as well as health care system resources for GenAI adoptions, the potential benefits of GenAI GLP-1/dual RA management tools may be inequitably distributed.

Bias within GenAI training datasets has the potential to reinforce existing inequities [17]. Conscious efforts are likely to be needed to evaluate and tailor model training data for the populations of interest and through comparing and validating different samples of training data for representativeness [34]. The development and use of frameworks to evaluate the impact of GenAI use on health disparities and guide model modifications, as explored in other areas such as clinical predictive modeling [35], may be useful for guiding the equitable use of GenAI in clinical care [36].

Correspondingly, the development of GenAI obesity medication management and other GenAI-driven clinical tools should engage equitable digital design philosophies such as liberatory design [37]. Practical outcomes of this may include integrating GenAI into current system technologies that are widely available, such as SMS text messaging or existing EHR platforms, thus allowing for greater accessibility to these tools. Furthermore, to serve as health equity promotion interventions themselves, GenAI tools could be designed to detect and address known structural inequities, thereby proactively mitigating potential conscious or unconscious biases from the HCP.

## Conclusions

The rapidly escalating use of GLP-1/dual RA medications is poised to overwhelm an already overburdened HCP workforce and health care delivery system, stifling its potentially dramatic benefits. Early adoption of GenAI to facilitate the management of these GLP-1/dual RAs has the potential to improve health outcomes while decreasing its concomitant workload. Investment in GenAI’s potential to support GLP-1/dual RA management is greatly needed. This effort should be guided by inclusive design principles and deep integration into clinical workflows to achieve scalable impact on clinical outcomes.

## Abbreviations

AI: artificial intelligence
EHR: electronic health record
GenAI: generative artificial intelligence
GLP-1: glucagon-like peptide 1
HCP: health care provider
RA: receptor agonist
